# Nomogram for the prediction of diabetic nephropathy risk among patients with type 2 diabetes mellitus based on a questionnaire and biochemical indicators: a retrospective study

**DOI:** 10.18632/aging.103259

**Published:** 2020-06-02

**Authors:** Yuhong Hu, Rong Shi, Ruohui Mo, Fan Hu

**Affiliations:** 1 School of Public Health, Shanghai University of Traditional Chinese Medicine, Shanghai, China

**Keywords:** diabetic nephropathy, predictors, nomogram, type 2 diabetes mellitus, risk factors

## Abstract

Purpose: Develop a diabetic nephropathy incidence risk nomogram in a Chinese population with type 2 diabetes mellitus.

Results: Predictors included systolic blood pressure, diastolic blood pressure, fasting blood glucose, glycosylated hemoglobin A1c, total triglycerides, serum creatinine, blood urea nitrogen and body mass index. The model displayed medium predictive power with a C-index of 0.744 and an area under curve of 0.744. Internal verification of C-index reached 0.737. The decision curve analysis showed the risk threshold was 20%. The value of net reclassification improvement and integrated discrimination improvement were 0.131, 0.05, and that the nomogram could be applied in clinical practice.

Conclusion: Diabetic nephropathy incidence risk nomogram incorporating 8 features is useful to predict diabetic nephropathy incidence risk in type 2 diabetes mellitus patients.

Methods: Questionnaires, physical examinations and biochemical tests were performed on 3489 T2DM patients in six communities in Shanghai. LASSO regression was used to optimize feature selection by running cyclic coordinate descent. Logistic regression analysis was applied to build a prediction model incorporating the selected features. The C-index, calibration plot, curve analysis, forest plot, net reclassification improvement, integrated discrimination improvement and internal validation were used to validate the discrimination, calibration and clinical usefulness of the model.

## INTRODUCTION

Over the past 20 years, due to the increase in the obesity rate and the prevalence of sedentary lifestyles, the number of people worldwide diagnosed with diabetes has been increasing [1], which is rapidly becoming a public health problem in both developed and developing countries [2]. According to the report released by the International Diabetes Federation in 2015, the prevalence of diabetes among adults worldwide is 9.1% [3]. According to this figure, 415 million adults worldwide have diabetes, and 318 million adults have impaired blood glucose regulation, composing a group with a high risk of diabetes [4]. It was estimated that the number of diabetes patients among adults aged 20 and over will increase from 171 million in 2000 to 552 million in 2030 [5], and this figure is expected to exceed 693 million in 2045 [6]; the most dramatic increase is estimated to occur in developing countries [7].

The lifestyle of Chinese people is also changing rapidly, leading to a continuous increase in diabetes incidence and the obesity rate. The diabetes prevalence rate was lower than 1% in 1980 [8], reaching 5.5% in 2001 [9], 9.7% in 2008 [10], and 10.9% in 2013 [11]. According to the latest international diagnostic criteria, 11.6% of Chinese adults had diabetes in 2010, the prevalence of prediabetes was estimated at 50.1% [12], and approximately 114 million people had type 2 diabetes mellitus (T2DM). China has become the country with the largest number of people with diabetes. Moreover, there is an obvious trend toward younger people being affected [13], and young people with diabetes have a higher risk of chronic complications, which is the main cause of the mortality and morbidity associated with diabetes [4].

Diabetic nephropathy (DN) is one of the most common microvascular complications of diabetes [14]. The incidence of DN in patients with T2DM is 20-40% [15], and DN increases the risk of death in patients with T2DM. DN is the reason 20% to 40% of patients with end-stage renal disease (ESRD) begin treatment. In Australia, the number of newly diagnosed T2DM patients who started dialysis increased fivefold from 1993 to 2007 [16]. In the United Kingdom, the prevalence of T2DM with DN among adults registered in the primary health care system increased from 11.5% to 15.1% from 2005 to 2007 [17]. In Malaysia, the prevalence of early T2DM with DN was 25.4% among hospital outpatients [18]. The DISS project [19] found that 25% of patients with T2DM developed DN during a 17-year follow-up in Sweden. In Europe, Kainz et al [20] predicted that ESRD due to DN would increase by 3.2% annually from 2012 to 2025. According to the third national health and nutrition survey in the United States, the standardized case fatality rate of patients with diabetes without renal disease is 11.5% and that of patients with diabetes combined with renal disease is 31.1% [21].

The incidence of DN in China has surpassed that of primary glomerulonephritis and has become the first cause of chronic renal insufficiency, the first cause of hospitalization for chronic kidney disease in urban regions and the second leading cause of ESRD [22]. It is estimated that the number of patients with diabetes-related chronic kidney disease in China has reached 24.3 million [22]. The number of DN-related deaths in the world also increased by 2.8-fold from 463000 in 1990 to 1731000 in 2013, which is the fastest increase among chronic diseases [23].

Most Chinese studies on DN have focused on inpatients in secondary and tertiary hospitals. For example, an investigation of 3469 inpatients with T2DM in Beijing, Shanghai, Tianjin, and Chongqing showed that the incidence of DN complications was 39.7% from 1991 to 2000. The prevalence of diabetic microvascular complications in large Chinese cities has reached or even exceeded the average level in developed countries.

Early diagnosis and treatment can reduce the mortality and disability rate of DN and improve the quality of life of patients. However, due to the insidious onset of DN, renal function damage is irreversible at the time of diagnosis of most patients. Therefore, the purpose of this study was to develop a valid and simple prediction tool using a questionnaire, physical examination, biochemical tests, and estimation for patients with type 2 diabetes mellitus (T2DM) to assess the risk of DN incidence [24].

## RESULTS

### Patient characteristics

All 3489 community-based patients with T2DM were divided into nondiabetic nephropathy (NDN) and DN groups according to the ratio of urinary microalbumin to uric creatinine (ACR), and 701 patients were diagnosed with DN (prevalence 20.10%). Among all participants, there were 1562 males (44.8%) and 1927 females (55.2%), the mean age was 64.54 ± 6.78 years [range 28–87 years], and the duration of disease was 9.48 ± 6.93 years. All data of patients, including demographics, lifestyle habits, physical examination results and biochemical test results, in the two groups are given in Table 1.

**Table 1:** Differences in demographic and clinical characteristics between the NDN and DN groups.

**Demographic characteristics**	**n (%)**		
	**NDN**	**DN**	**Total (n=3489)**
Sex			
Male	1226 (44.0)	336 (47.9)	1562 (44.8)
Female	1562 (56.0)	365 (52.1)	1927 (55.2)
Age(years)			
<50	57 (2.0)	6 (0.9)	63 (1.8)
50-69	2102 (75.4)	486 (69.3)	2588 (74.2)
≥70	629 (22.6)	209 (29.8)	838 (24.0)
Disease duration (years)			
<5	795 (28.5)	148 (21.1)	943 (27.0)
5-9	791 (28.4)	187 (26.7)	978 (28.0)
10-14	589 (21.1)	166 (23.7)	755 (21.6)
15-19	378 (13.6)	119 (17.0)	497 (14.2)
20-24	157 (5.6)	53 (7.6)	210 (6.0)
25-29	50 (1.8)	14 (2.0)	64 (1.8)
≥30	28 (1.0)	14 (2.0)	42 (1.2)
**Lifestyle habits**			
Smoking			
No	2070 (74.2)	527 (75.2)	2597 (74.4)
Yes	718 (25.8)	174 (24.8)	892 (25.6)
Consuming alcohol			
No	2078 (74.5)	531 (75.7)	2609 (74.8)
Yes	710 (25.5)	170 (24.3)	880 (25.2)
**Physical examination results**			
SBP (mmHg)			
<130	713 (25.6)	72 (10.3)	785 (22.5)
130-139	585 (21.0)	111 (15.8)	696 (19.9)
≥140	1490 (53.4)	518 (73.9)	2008 (57.6)
DBP (mmHg)			
<80	1252 (45.1)	220 (31.4)	1472 (42.0)
80-89	1013 (36.3)	286 (40.8)	1299 (37.2)
≥90	523 (18.8)	195 (27.8)	718 (20.6)
BMI (kg/m^2^)			
<24	1038 (37.2)	161 (23.0)	1199 (34.4)
24-27.99	1248 (44.8)	340 (48.5)	1588 (45.5)
≥28	502 (18.0)	200 (28.5)	702 (20.1)
WHR			
<0.9	1269 (45.5)	251 (35.8)	1520 (43.6)
≥0.9	1519 (54.5)	450 (64.2)	1969 (56.4)
**Biochemical tests result characteristics**			
FBG (mmol/L)			
<6.1	682 (24.5)	26 (3.7)	708 (20.3)
6.1-6.9	554 (19.9)	126 (18.0)	680 (19.5)
>6.9	1552 (55.7)	549 (78.3)	2101 (60.2)
PBG (mmol/L)			
<7.8	593 (21.3)	64 (9.1)	657 (18.8)
7.8-11.0	748 (26.8)	157 (22.4)	905 (25.9)
>11.0	1447 (51.9)	480 (68.5)	1927 (55.2)
HbA1c (%)			
<6	467 (16.8)	46 (6.6)	513 (14.7)
6-6.9	1054 (37.8)	229 (32.7)	1283 (36.8)
>6.9	1267 (45.4)	426 (60.8)	1693 (48.5)
TC (mmol/L)			
<4.5	1091 (39.1)	217 (31.0)	1308 (37.5)
≥4.5	1697 (60.9)	484 (69.0)	2181 (62.5)
TGs (mmol/L)			
<1.7	1615 (57.9)	317 (45.2)	1932 (55.4)
≥1.7	1173 (42.1)	384 (54.8)	1557 (44.6)
LDL (mmol/L)			
<1.8	1889 (67.8)	451 (64.3)	2340 (67.1)
≥1.8	899 (32.2)	250 (35.7)	1149 (32.9)
HDL (mmol/L)			
<1.0	104 (3.7)	14 (2.0)	118 (3.4)
≥1.0	2684 (96.3)	687 (98.0)	3371 (96.6)
BUN (mmol/L)			
<3.2	110 (3.9)	4 (0.6)	114 (3.3)
3.2-7.1	2300 (82.5)	549 (78.3)	2849 (81.7)
>7.1	378 (13.6)	148 (21.1)	526 (15.1)
SCR (μmol/L)			
<44	100 (3.6)	26 (3.7)	126 (3.6)
44-106	2646 (94.9)	645 (92.0)	3291 (94.3)
>106	42 (1.5)	30 (4.3)	72 (2.1)
UA (μmol/L)			
<150	22 (0.8)	0 (0)	22 (0.6)
150-440	2602 (93.3)	681 (97.1)	3283 (94.1)
>440	164 (5.9)	20 (2.9)	184 (5.3)
UCR (mmol/L)			
<5.3	540 (19.4)	143 (20.4)	683 (19.6)
5.3-18.0	2166 (77.7)	543 (77.5)	2709 (77.6)
>18.0	82 (2.9)	15 (2.1)	97 (2.8)
UMA (mg/L)			
<30	2522 (90.5)	88 (12.6)	2610 (74.8)
≥30	266 (9.5)	613 (87.4)	879 (25.2)
ACR (mg/g)			
<30	2788 (100)	0 (0)	2788 (79.9)
30-300	0 (0)	676 (96.4)	676 (19.4)
≥300	0 (0)	25 (3.6)	25 (0.7)

### Feature selection

**Figure 1:**
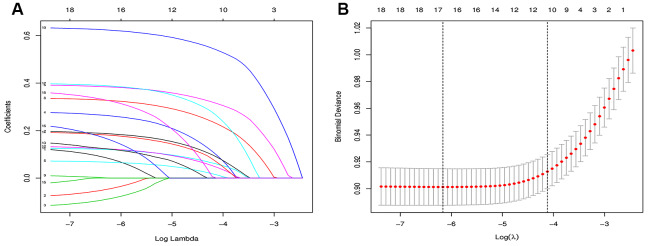
**Feature selection using the LASSO binary logistic regression model.** (**A**) Features selection by LASSO binary logistic regression model. By verifying the optimal parameter (lambda) in the LASSO model, the partial likelihood deviance (binomial deviance) curve was plotted versus log(lambda). Dotted vertical lines were drawn based on 1 SE of the minimum criteria (the 1-SE criteria). (**B**) Features selection by LASSO binary logistic regression model. A coefficient profile plot was produced against the log(lambda) sequence in figure 1(A). 16 features with nonzero coefficients were selected by optimal lambda.

**Figure 2:**
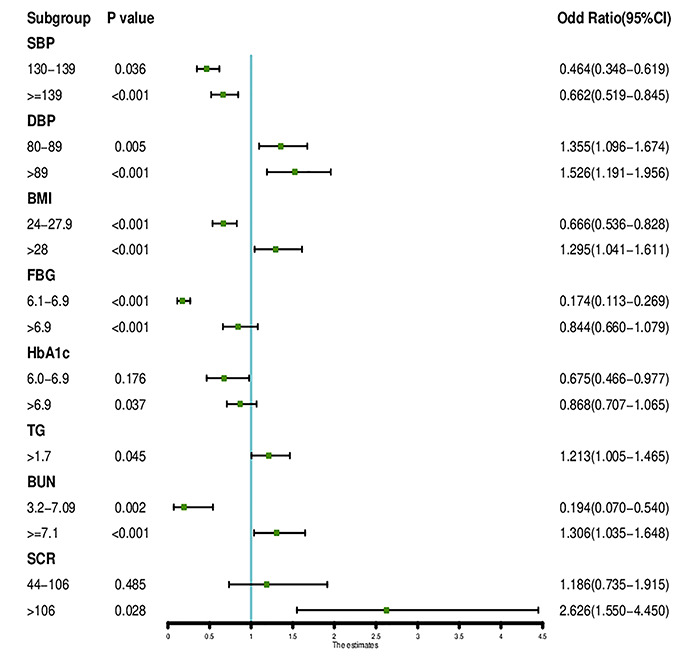
**The forest plot of the OR of the selected feature.** Use of forest plot for outcome in LASSO regression model and logistic regression analysis.

Of the demographics, lifestyle habits, physical examination results and biochemical test results, 19 features were reduced to 8 potential predictors on the basis of the results of 3489 patients (~2:1 ratio; Figures 1, 2) and had nonzero coefficients in the least absolute shrinkage and selection operator (LASSSO) regression model. The coefficient of lambda was 0.002. These features included systolic blood pressure (SBP), diastolic blood pressure (DBP), fasting blood glucose (FBG), glycosylated hemoglobin A1c(HbA1c), total triglycerides (TGs), serum creatinine (SCR), blood urea nitrogen (BUN) and body mass index (BMI) (Table 2).

**Table 2:** Predictive factors for DN incidence risk in T2DM patients

**Intercept and variable**	**Prediction model**					
	**β**	**z-value**	**P-value**	**OR**	**2.5% CI**	**97.5% CI**
Intercept	-7.174	-8.97	<0.001***	0.001	0	0.003
SEX=Male	0.123	1.12	0.264	1.131	0.911	1.405
AGE=50-69	0.532	1.17	0.241	0.587	0.241	1.430
AGE=>=70	0.813	1.77	0.078	1.325	1.083	1.621
COURSE=5-9	-0.010	-0.07	0.942	1.010	0.781	1.305
COURSE=10-14	0.101	0.730	0.464	1.116	0.869	1.434
COURSE=15-19	0.225	1.48	0.139	1.265	0.957	1.672
COURSE=20-24	0.294	1.46	0.144	1.355	0.930	1.975
COURSE=25-29	0.075	0.22	0.823	1.089	0.568	2.086
COURSE=>=30	0.653	1.77	0.077	1.940	0.947	3.973
SMOKE=YES	-0.062	-0.49	0.628	0.939	0.730	1.209
DRINK=YES	-0.131	-1.05	0.293	0.877	0.730	1.209
SBP=130-139	0.355	2.10	0.036*	0.464	0.348	0.619
SBP>139	0.767	5.22	<0.001***	0.662	0.519	0.845
DBP=80-89	0.304	2.81	0.005**	1.355	1.096	1.674
DBP=>89	0.443	3.34	<0.001***	1.526	1.191	1.956
BMI=24-27.9	0.406	3.65	<0.001***	0.666	0.536	0.828
BMI=>=28	0.665	5.16	<0.001***	1.295	1.041	1.611
FBG=6.1-6.9	1.578	6.88	<0.001***	0.174	0.113	0.269
FBG=>6.9	1.748	7.89	<0.001***	0.844	0.660	1.079
PBG=7.8-11.0	0.194	1.14	0.256	0.740	0.539	1.015
PBG=>11.0	0.302	1.87	0.062	0.898	0.716	1.125
HbA1c=6.0-6.9	0.252	1.35	0.176	0.675	0.466	0.977
HbA1c>6.9	0.393	2.08	0.037*	0.868	0.707	1.065
TC=>=4.5	0.137	1.32	0.187	0.872	0.711	1.069
TGs=>=1.7	0.193	2.01	0.045*	1.213	1.005	1.465
HDL=>=1.0	0.229	0.73	0.468	0.795	0.429	1.476
BUN=3.2-7.09	1.639	3.14	0.002**	0.194	0.070	0.540
BUN=>=7.1	1.906	3.59	<0.001***	1.306	1.035	1.648
SCR=44-106	-0.1707	-0.70	0.485	1.186	0.735	1.915
SCR=>106	0.7949	2.20	0.028*	2.626	1.550	4.450

Note: β is the regression coefficient. “***” indicates P<0.001, “**” indicates P<0.01, “*” indicates P<0.05.

### Development of an individualized prediction model

**Figure 3:**
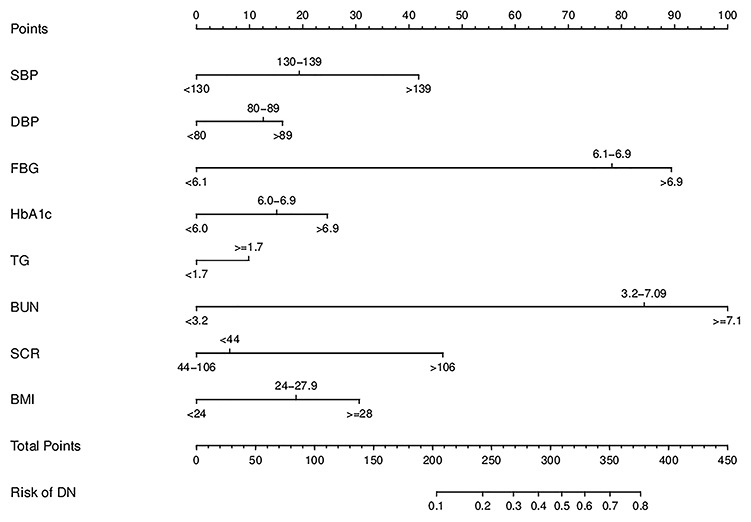
**Developed DN incidence risk nomogram.** The DN incidence risk nomogram was developed in the array, with SBP, DBP, FBG, HbA1c, TG, SCR, BUN and BMI incorporated.

The results of the logistic regression analysis among SBP, DBP, FBG, HbA1c, TGs, SCR, BUN and BMI are given in Table 3. A model that incorporated the above independent predictors was developed and presented as the nomogram (Figure 3).

**Table 3:** C-index in the array

**C-index (95% CI)**	**Dxy**	**aDxy**	**variance**	**Z-value**	**P-value**	**n**
0.744 (0.7242, 0.764)	0.488	0.488	0.02	24.74	0	3489

### Apparent performance of the DN incidence risk nomogram in the array

**Figure 4:**
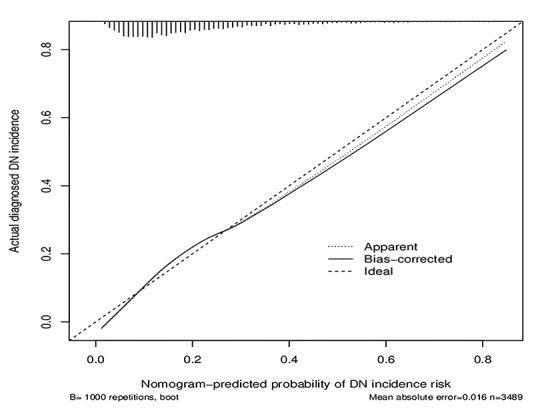
**Calibration curves of the DN incidence risk nomogram prediction in the cohort.** The x-axis represents the predicted DN incidence risk. The y-axis represents the actual diagnosed DN. The diagonal dotted line represents a perfect prediction by an ideal model. The solid line represents the performance of the nomogram, of which a closer fit to the diagonal dotted line represents a better prediction.

**Figure 5:**
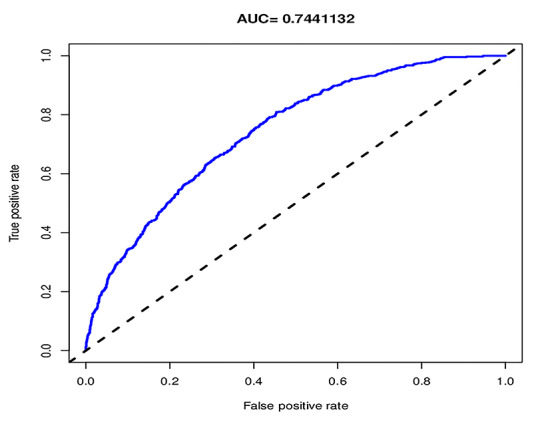
**The pooled AUC of the ROC curve.** The y-axis means the true positive rate of the risk prediction. The x-axis means the false positive rate of the risk prediction. The blue line represents the performance of the nomogram.

For the 3489 T2DM patients in the array, the calibration curve of the nomogram to predict DN risk showed medium agreement (Figure 4). The C-index of the nomogram was 0.744 (95% CI: 0.724–0.764), and the C-index of internal verification was 0.737, which indicated that the model had medium prediction accuracy. The area under the receiver operating characteristic curve (AUC) was 0.744 (Figure 5), and the cutoff was 0.14, which indicates moderate performance. To summarize the results from the above verification, the nomogram of the model has medium prediction ability.

### Clinical use

**Figure 6:**
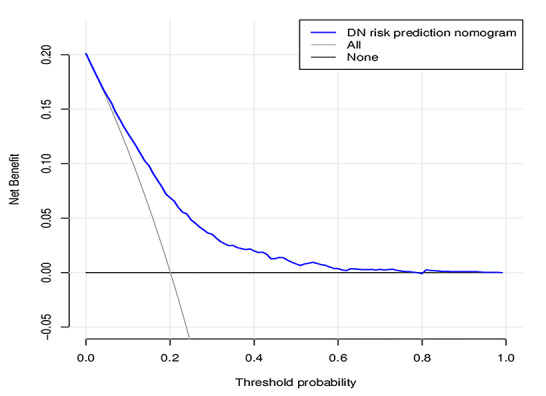
**Decision curve analysis for the DN incidence risk nomogram.** The y-axis measures the net benefit. The dotted line represents the DN incidence risk nomogram. The thin solid line represents the assumption that all patients are diagnosed as DN. Thin thick solid line represents the assumption that no patients are diagnosed as DN. The decision curve showed that if the threshold probability of a patient and a doctor is >20%, respectively, using this DN incidence risk nomogram in the current study to predict DN incidence risk adds more benefit than the intervention-all-patients scheme.

The decision curve analysis for the DN incidence risk nomogram is presented in Figure 6. The decision curve showed that if the threshold probability of a patient and a doctor is >20%, using this DN incidence nomogram to predict DN incidence risk for T2DM patients is more beneficial than the existing scheme. Within this range, the net benefit was comparable with several overlaps on the basis of the DN incidence risk nomogram.

In this study, according to AUC 0.744, cutoff 0.14, calibration, and decision curve analysis, the predictive ability of the nomogram graph needed to be further verified, so net reclassification improvement (NRI) and integrated discrimination improvement (IDI) verifications were introduced, and 8 characteristic indicators SBP, DBP, FBG, HbA1c, TGs, BUN, SCR, and BMI were selected by LASSO and logistic regression. Moreover, the above indicators are clinical indicators of DN that were included in the nomogram.

**Figure 7:**
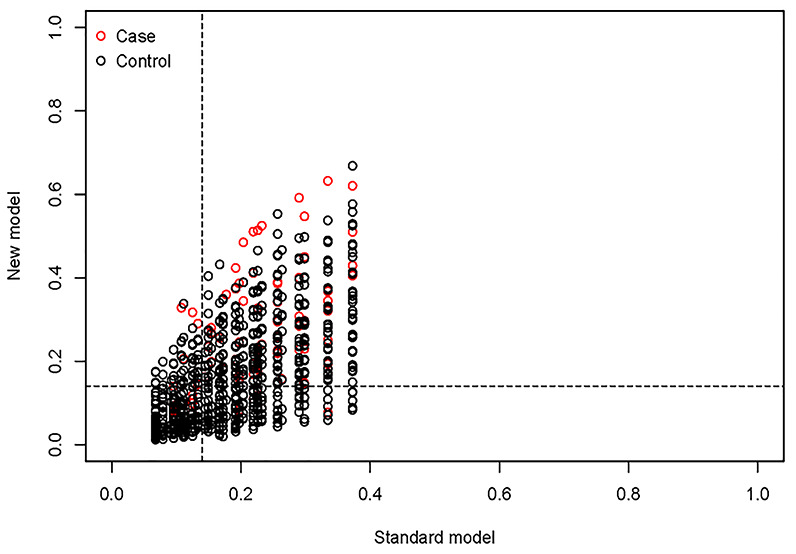
**Model comparison based on NRI.** The value of cutoff is 0.14, the value of NRI is 0.131 (0.086, 0.168).

According to the above indicators, model A, with only physical examination indicators, and model B, with all indicators, were established. Model A includes SBP, DBP, BMI, and model B includes SBP, DBP, FBG, HbA1c, TGs, BUN, SCR, and BMI. The models were verified through NRI and IDI. Model B was compared with model A. The NRI was calculated for categorical variables. The cut-off value was 0.14, the value of NRI was 0.131 (Figure 7), and the value of IDI was 0.05 (Table 4). Model B was better than model A, according to both NRI and IDI. The verification proves that Model B has a certain predictive ability in this study.

**Table 4:** Comparison of the predictive abilities of different models using NRI and IDI.

	**Model A~B (n=3489)**	
	**NRI (0.131)**	**IDI (0.051)**
P	0	0
2.5% CI	0.086	0.043
97.5% CI	0.168	0.058

**Figure 8:**
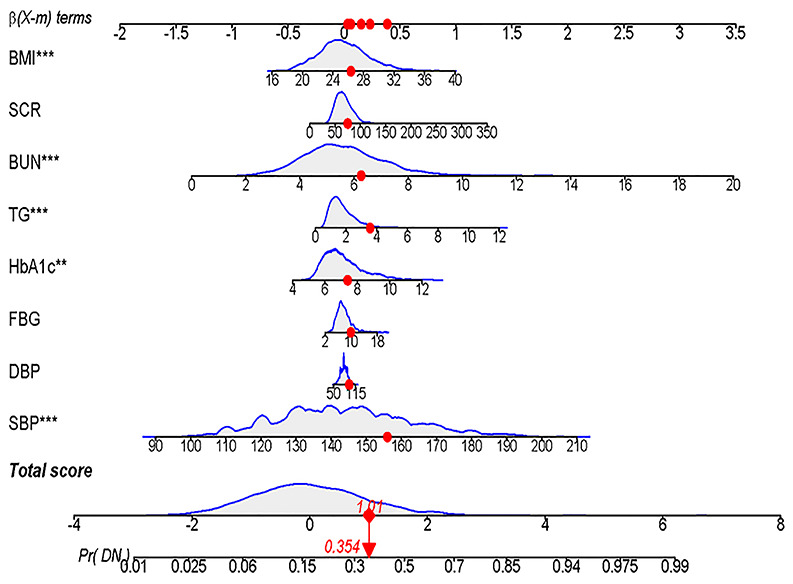
**Dynamic Nomogram.** A T2DM patient was randomly selected from the population, and the DN incidence of the patient was predicted based on the 8 characteristic indicators of the nomogram.

According to the characteristic indicators selected by the model, a patient is randomly selected from the population. The patient indicators are as follows: SBP=156, DBP=96, FBG=9.9, HBA1C=7.4, TGs=3.57, BUN=6.26, SCR=75, BMI=26.35. A dynamic nomogram is established to predict the incidence of DN (Figure 8). Therefore, the team developed a dynamic nomogram online APP to predict the DN of T2DM, the URL: https://doctorhu.shinyapps.io/DN_DynNomapp/. The APP is developed based on the characteristic indicators derived from the model. It can assist clinicians in diagnosing DN and can also be used in community-based diabetes DN prevention [25].

## DISCUSSION

Currently, nomograms are widely used as prognostic devices in oncology and medicine [26]. Nomograms depend on user-friendly digital interfaces, have improved accuracy, and provide more easily understood prognoses to aid better clinical decision making [27]. Our study was the first to apply a nomogram to the incidence of DN in T2DM patients.

We developed and validated a novel prediction tool for DN incidence risk among T2DM patients using eight easily available variables. Incorporating demographics, lifestyle habits, physical examination results and biochemical test results into an easy-to-use nomogram facilitates the prediction of DN incidence among T2DM patients. Internal validation in the cohort demonstrated medium discrimination and calibration power, and the medium C-index value in the internal validation indicated that this nomogram can be widely and accurately used due to the large sample size [27].

### Prevalence of DN among T2DM patients is consistent with other studies

In our study, the prevalence of DN in community-based patients with T2DM was 20.10%, which was consistent with some other studies [15].

According to a prospective survey of 66726 patients with T2DM in 28 countries, the prevalence of DN was 27.9%, with the highest prevalence in the Middle East and Russia and the lowest prevalence in South Asia [28]. In the United States, the National Health and Nutrition Survey showed that the prevalence of DN in patients with diabetes is 23.7% [29, 30]. In the United Kingdom Prospective Diabetes Study (UKPDS) [31], 2% to 3% of newly diagnosed T2DM patients develop DN every year. In South Korea, the National Health and Nutrition Survey in 2011 [32] found that 26.7% of patients with diabetes had DN, and 4.7% of them had progressed to clinical nephropathy. Statistics from European countries show that approximately 20% of diabetic patients can develop DN, which is the most serious complication of diabetes and one of the main causes of dialysis in diabetic patients [33].

However, current Chinese studies have mainly focused on inpatients, while few studies have included community-based epidemiological surveys, with even fewer large-scale studies of community patients with T2DM and DN. A retrospective investigation of chronic complications of T2DM by the Chinese Diabetes Society in 2001 showed that the prevalence of renal complications was 34.7% in inpatients with T2DM in third-class hospitals.

### Main risk factors for DN in T2DM patients

In the risk factor analysis, SBP, DBP, FBG, HbA1c, TGs, SCR, BUN and BMI were associated with DN incidence in T2DM patients. This nomogram suggested that lower SBP, DBP, FBG, HbA1c, TGs, SCR, BUN and BMI may be the key factors that can reduce the DN incidence risk of T2DM patients.

### Systolic blood pressure and diastolic blood pressure

SBP and DBP are the main diagnostic indicators of hypertension. This study found that SBP and DBP are risk factors for DN in patients with T2DM. It has been proven that among T2DM patients, hypertension is an independent risk factor for the occurrence and development of microvascular complications [34]. The prevalence of hypertension is higher in patients with DN. SBP and DBP were significantly higher in DN patients than in NDN patients (P <0.001). Boguslawa et al [35] found that for every 10 mmHg increase in blood pressure, the risk of microalbuminuria (MAU) in T2DM patients would increase by 23%. In Thailand, a nationwide cross-sectional study including 55797 T2DM patients found that patients with hypertension had a 1.32-fold higher risk of microangiopathy than patients with normal blood pressure [36]. A European study of T2DM patients [37] found that the SBP and DBP in patients with MAU were significantly higher, and multivariate analysis showed that SBP was an independent risk factor for MAU in male patients with diabetes. In the UKPDS, a randomized clinical trial including 1148 T2DM patients [38], it was found that strict control of blood pressure could reduce the risk of microangiopathy by 37% (P=0.0092). A study conducted in Turkey with 202 T2DM patients in Zonguldak Atatürk State Hospital found that SBP (P=0.043) was independently related to logarithmically converted 24-hour UAER [39]. Leehey et al [40] confirmed that DN patients with mean SBP ≥140 mmHg had poor renal prognosis.

The cause of hypertension in diabetes mellitus patients may be associated with DN pathogenesis with small artery pressure causing renal arteriolar sclerosis, hemal wall thickening, luminal narrowing, increased glomerular filtration (increased plasma osmotic pressure during hyperglycemia, which promotes the entry of interstitial fluid into the blood vessels), which can directly lead to the presence of hyperfiltration (increased the glomerular filtration rate (GFR), and there is no accepted GFR standard yet) and therefore polyuria and hyperperfusion (increased plasma flow). The major early changes in DN include glomerulosclerosis, and hyperglycemia puts blood vessels in a hyperperfusion state. Moreover, capillary blood pressure is increased, and filtration pressure is increased, resulting in increased GFR, glomerular hyperperfusion and excessive filtration. Mesangial cells expand, epithelial cell foot processes fuse and produce dense droplets, and glomerular epithelial cells fall off the basement membrane, leading to glomerular fibrosis [41]. Small and medium-sized artery endothelial cells and smooth muscle cells undergo secondary hardening and damage. Hyperplasia after repair causes thickening of the basement membrane and high levels of glucagon in people with diabetes lead to the activation of the renin-angiotensin system (RAS) to speed up diabetic microvascular and major vascular complications, eventually leading to kidney damage and renal dysfunction [42]. Therefore, effective blood pressure reduction can significantly reduce the occurrence and development of DN [43], reduce the excretion rate of urinary albumin [44], and delay the occurrence of decompensated nephropathy. DN is occult, and there is no obvious decline in renal function in the early stage, but there has been a state of high perfusion and high filtration of the glomeruli, and damage to the glomeruli has begun [45, 46].

### Serum creatinine and blood urea nitrogen

SCR is the most commonly used index to assess whether the kidney is damaged. When the glomerulus is severely damaged and the GFR is decreased, SCR is increased [47]. However, because of the kidney's strong ability to compensate and form reserves, SCR remains at a normal level in the early stage of kidney injury; then, SCR increases rapidly and significantly, but SCR is also affected by sex, muscle mass and diet [48].

As the main nitrogen-containing substance excreted by the kidney, BUN can reflect the filtration function of the glomerulus to some extent, which is of great significance to the condition, course and prognosis of kidney disease. It was clearly proven that glomerular damage, tubular injury, inflammatory responses, and oxidative stress contribute to the development of DN.

### Body mass index and total triglycerides

High BMI and TGs indicate that patients are obese, and such patients tend to have high blood lipid levels. Increased blood TGs will increase blood viscosity, thus reducing blood flow velocity and inducing microvascular lesions. Under the condition of high glucose, cells will become hyperosmotic, and a large amount of extracellular fluid infiltration will cause cell edema, destroy cell structure, and directly or indirectly inhibit the function of glomerular and tubular cells. The hyperfiltration state of the glomerulus is a direct factor leading to proteinuria. Moreover, the metabolic demand of the kidney in obese people is higher than that of nonobese people. Both anthropometric and biochemical indices of adiposity and, to a smaller extent, elevated blood pressure were implicated as major determinants of glomerular hyperfiltration [49, 50]. To meet the higher metabolic demand, renal glomerular arterioles continue to expand, blood perfusion continues to increase, GFR increases, and the kidney's automatic regulation ability is impaired. Simultaneously, the pressure in glomerular capillaries increases, the tension of the tube wall increases, and the capillaries become thicker, making the distribution of podia cells relatively sparse, leading to the decrease in renal function [51]. Obesity further increases the risk of DN and ESRD [52].

It was found in the DCCT/EDIC study that lower TGs were associated with a reduced risk for progression from MAU to clinical albuminuria (CAU) or ESRD [53]. A cross-sectional epidemiological study in Singapore [54] showed that obesity significantly increased the risk of DN, with the risk ratios for DN in groups with a BMI 23-24.9, 25-29.9 and ≥ 30 being 3.12, 2.49 and 3.70 (95% CI: 2.13~6.42), respectively, compared with the group with a BMI <23. Cohen et al [55] further confirmed that overweight and obesity are risk factors for chronic kidney disease (CKD). The multivariate model analysis of Kwan JM et al [56] found that obese patients had a significantly increased risk of adverse transplant outcomes, including delayed graft function recovery, graft failure, urinary protein and acute rejection. Hyperleptinemia caused by obesity is also involved in the occurrence of DN: leptin can stimulate the proliferation of glomerular endothelial cells and glomerular mesangial cells, as well as the proliferation of tubulointerstitial cells and matrix components, by inducing oxidative stress of glomerular endothelial cells, leading to the occurrence of glomerular sclerosis. In addition, obesity increases sympathetic excitability and hyperactivity of the RAS, which is also associated with the occurrence of DN. The Look AHEAD Research Group [57] found that weight loss, namely, calorie restriction and increased exercise, could significantly reduce the occurrence and development of DN in obese or overweight T2DM patients. A randomized controlled study in Hong Kong showed that effective comprehensive management of T2DM patients could effectively reduce the risk of ESRD and death [58].

### Fasting blood glucose and glycosylated hemoglobin A1c

FBG is the most basic indicator of the development of T2DM. HbA1c can basically reflect the average blood glucose level in the past two to three months, and long-term hyperglycemia results in protein nonenzymatic glycosylation, leading to systemic microvascular damage and the promotion and development of microangiopathy [59]. It was confirmed that strict glycemic control can significantly reduce the risk of diabetic microvascular complications [60]. Epidemiological analysis of the Diabetes Control and Complications Trail [61] showed that for every 10% reduction in HbA1c, there was a 25% and 44% reduction in the risk of MAU and CAU, respectively, in patients with type 1 diabetes mellitus. Similarly, the UKPDS showed that for every 1% decrease in HbA1c, there was a 37% reduction in the risk of T2DM microvascular complications. The UKPDS was a clinical study that included 10 years of blood glucose intervention in patients with newly diagnosed T2DM [62] and showed that HbA1c (7.0%) in the intervention group was 11% lower than that in the traditional treatment group (7.9%), and the risk of microvascular complications was reduced by 25%. The incidence of DN was reduced by 33% at the 12-year follow-up. Ten years after the trial, the benefits of intensive glycemic control persisted after treatment [35]. Moreover, a study in Taiwan found that HbA1c significantly promoted DN progression in T2DM patients with high levels of normal albuminuria [63]. Moreover, a meta-analysis showed that patients with HbA1c<7% had less MAU than patients with HbA1c<7.9% [64]. Chronic hyperglycemia in diabetic patients accelerates nonenzymatic glycosylation, leading to increased hypoxia of tissues and the release of vasoactive substances, which result in hemodynamic changes and are important risk factors for DN. The effect of blood glucose on DN is reflected in three aspects. The first is poor long-term blood glucose control. Ficociello et al [65] conducted a 10-year follow-up study and found that diabetic patients with persistent hyperglycemia or poor blood glucose control had a 3 to 8 times higher risk of DN development or progression. Persistent hyperglycemia can induce glomerular mesangial hyperplasia, resulting in basement membrane thickening and glomerular capillary wall sclerosis, ultimately leading to the occurrence of proteinuria. On the other hand, persistent hyperglycemia causes the glomerulus to be in a state of hyperperfusion and hyperfiltration, resulting in capillary sclerosis and changes in vascular permeability, which ultimately leads to plasma protein deposition on capillary walls, progressive vascular hyperplasia, hyaline degeneration and intravascular thrombosis [66]. Second, blood glucose fluctuates greatly. The UKPDS report confirmed that blood glucose fluctuation was closely related to microvascular complications and was a risk factor for the occurrence or progression of DN, independent of blood glucose and HbA1c, even exceeding the risk associated with hyperglycemia alone [67]. A newly published study confirmed that fasting glucose variation and HbA1c variation are risk factors for progression to ESRD among patients with diabetes [68]. Moreover, studies have shown that not only hyperglycemia but also insulin resistance or hyperinsulinemia before the onset of diabetes begins to result in glomerular hypertrophy and thickened capillary basement membranes [69].

### Limitations

There are several limitations in our current study. First, our acquired data might have a low representation of males and only a partial representation of T2DM patients. The cohort was not representative of all Chinese patients with T2DM. Our study mainly focused on community-based T2DM patients in Shanghai, and patients who did not participate in community T2DM management were not included. The number of males and females included in our study was quite different. Second, risk factor analysis did not include all potential factors that affect DN incidence. Some possible aspects of DN incidence risk were not thoroughly assessed, such as drug treatment, exercise and other factors. Third, although the robustness of our nomogram was examined extensively with internal validation in the same population, there is a lack of external verification in other T2DM populations in other regions and countries. The nomogram needs to be externally evaluated in wider T2DM populations.

## CONCLUSIONS

This study developed a novel nomogram with a relatively moderate accuracy to help clinicians access the risk of DN incidence in T2DM patients. With an estimate of individual risk, clinicians and patients can take more necessary measures in regard to lifestyle monitoring and medical interventions. This nomogram requires broader external clinical validation, and further research is needed to determine whether individual interventions based on this nomogram will reduce DN incidence risk and improve treatment outcomes.

The prevalence rate of DN was 20.10% among community-based patients with T2DM in Shanghai, which was consistent with previous research. We found that HbA1c was one of the most important risk factors for DN, which suggests that the government should establish more effective and extensive DN screening and T2DM management programs at the community level, with special attention to interventions and monitoring of risk indicators, such as HbA1c, FBG, and hypertension. Moreover, patients should strengthen their self-management of T2DM, especially those with hypertension and obesity, and T2DM patients need to pay close attention to the occurrence of DN.

## MATERIALS AND METHODS

### Patients

The project started in September 2014 and was led by Professor Rong Shi, School of Public Health, Shanghai University of Traditional Chinese Medicine, and was funded and supported by the Shanghai Health and Health Commission for a five-year cohort study since September 2014. The research cooperation units include Ruijin Hospital Affiliated to Shanghai Jiaotong University, Shanghai Jiaotong University School of Medicine, Community Central Hospital Affiliated to Shanghai University of Traditional Chinese Medicine (including Daqiao Community, Jinyang Community, Yinhang Community, Siping Community, Sanlin Community, and Huamu Community).

**Figure 9:**
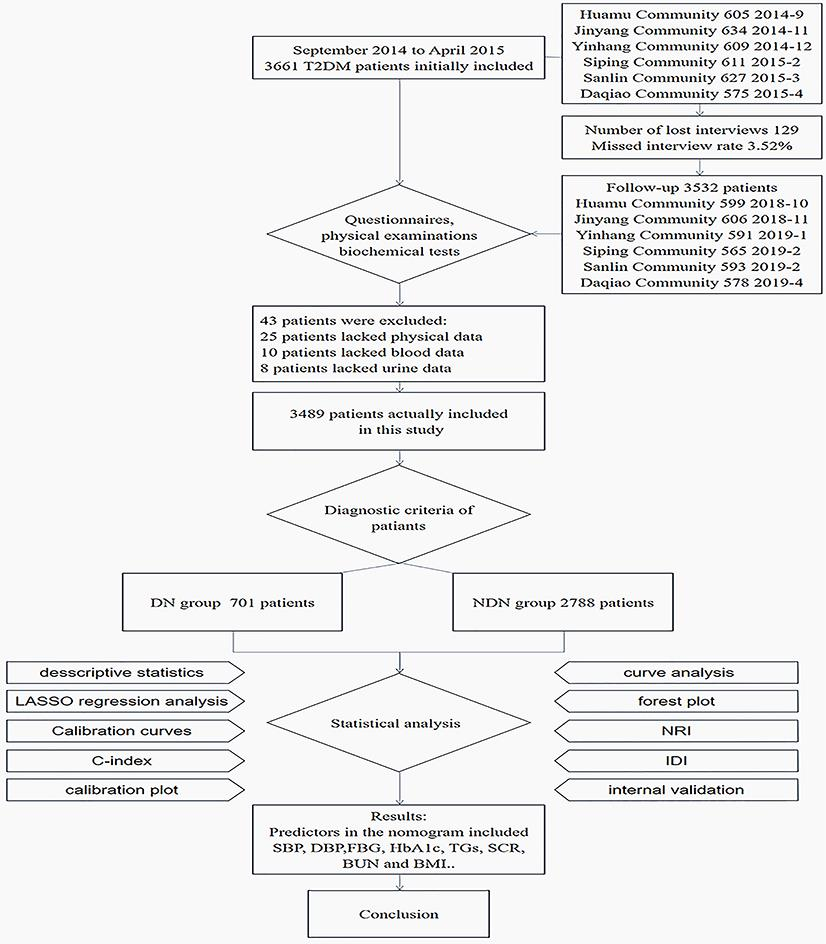
**Schematic diagram of research flow.** The research design, research object, research method and results are presented simply by flow chart.

The patients included in the study were diagnosed with T2DM in the hospital through medical records and medical history and participated in community diabetes management. The criteria defined by the World Health Organization in 1999 were used as the diagnostic criteria for diabetes [70]. The baseline data of this paper were collected from September 2014 to April 2015. The follow-up data of this paper were collected from October 2018 to April 2019. We obtained written informed consent from all participants before they were included in the study. A total of 3661 T2DM patients were enrolled in the study in the baseline survey. The inclusion criteria were as follows: patients with T2DM who were no more than 75 years old, had a district household registration or were a permanent resident (living in the community for at least 6 months), did not have serious chronic diseases (according to Chinese medical insurance regulations: malignant tumor, uremia dialysis, anti-rejection immunomodulator after organ transplant, chronic pulmonary heart disease, active tuberculosis, neurological deficit caused by sequelae of cerebrovascular disease, myocardial infarction/plug, chronic moderate/severe viral hepatitis, stage III high-risk and very-high-risk hypertension, chronic aplastic anemia, systemic lupus erythematosus, schizophrenia, hemophilia), and participated in the voluntary screening of diabetic complications. Among all participants, 43 were excluded due to incomplete data, and 129 were lost, the missed interview rate was 3.52%; finally, 3489 participants were included in the analysis. From September 2014 to April 2019, a total of 3489 T2DM patients in 6 communities were obtained baseline and follow-up data, and the final DN was used as the gold standard (Figure 9).

### Investigation methods

We determined the number of patients with T2DM in each community through the health center hospital and community health service center of Shanghai University of Traditional Chinese Medicine and invited the patients to the community service center for examination one week in advance and examined 100 patients per day. We completed the questionnaire survey, physical examination, and the collection of fasting blood, postprandial blood and urine for each patient. At seven o’clock in the morning, the patients' fasting blood was collected, and breakfast was provided. Two hours later, the patients' postprandial blood and urine were collected. During the waiting period, face-to-face questionnaires and physical examinations were administered, and participants were prompted by well-trained investigators to sign patient consent and provide information. The questionnaire survey included basic sociodemographic information, diabetes mellitus status (course of disease, complications, DN), and daily lifestyle habits (smoking, alcohol consumption). The physical examination included height, weight, waist circumference, hip circumference, SBP and DBP. BMI and waist-to-hip ratio (WHR) were calculated. On the day of the examination, blood and urine samples of all patients involved were sent to the laboratory for further processing. The blood samples were separated from the plasma and serum by centrifugation and then sent to a testing center for biochemical testing to obtain corresponding data. The laboratory examination included FBG, postprandial blood glucose (PBG), HbA1c, low-density lipoprotein (LDL), high-density lipoprotein (HDL), TGs, total cholesterol (TC), SCR, BUN, uric acid (UA), uric creatinine (UCR), and urinary microalbumin (UMA). ACR was calculated.

The diagnosis of DN was divided into three stages according to ACR (ACR less than 30 mg/g was defined as nonalbuminuria (NAU), 30 mg/g < ACR < 300 mg/g was defined as MAU, and ACR greater than or equal to 300 mg/g was defined as CAU [71]. Both MAU and CAU indicated DN in this study.

### Statistical analysis

All data, including demographics, lifestyle habits, and data from physical examinations and biochemical tests, are expressed as frequencies and counts (%). Statistical analysis was performed using R software (version 3.6.2; https://www.R-project.org). LASSO method is a shrinkage and variable selection method for linear regression models and is used to simplify high dimensional data [72]. To obtain the subset of predictors, LASSO regression analysis minimizes the prediction error for a quantitative response variable by imposing a constraint on the model parameters that cause regression coefficients for some variables to shrink toward zero [73]. Variables with a regression coefficient equal to zero after the shrinkage process are excluded from the model, while variables with nonzero regression coefficient variables are most strongly associated with the response variable. Based on the type measure of -2 log-likelihood and binomial family, the LASSO regression analysis running in R software runs 10 times K cross-validation for the centralization and normalization of included variables and then picks the best lambda value [74]. “Lambda.lse” gives a model with good performance but the least number of independent variables. Therefore, the LASSO method was used to select the optimal predictors from the present risk factors from the T2DM patients in the study. Then, multivariable logistic regression analysis was used to build a prediction model by introducing the features selected in the LASSO regression model [75]. Additionally, the forest plot was drawn to describe the P-value, OR and 95% CI of selected validation visually. The statistical significance levels were all two-sided. By introducing all the selected features and analyzing the statistical significance levels of the features, the statistically significant predictors were applied to develop a model of DN incidence risk prediction for patients with T2DM [76].

Calibration curves were used to evaluate the calibration of the DN incidence risk nomogram, accompanied by the Hosmer-Lemeshow test. A significant test statistic implies that the model does not calibrate perfectly [77]. To quantify the predictive ability of the nomogram, the C-index measurement was performed, and internal validation was performed using bootstrapping. Bootstrap samples are random samples drawn with replacement from the original sample, and 1000 bootstraps were repeatedly fitted in the model to evaluate the performance [78]. The receiver operating characteristic curve and the area under the receiver operating characteristic curve was used to perform medium discrimination of the quality of the risk nomogram to separate true positives from false positives [79]. Decision curve analysis was used to determine the clinical practicability of nomograms based on the net benefit according to different threshold probabilities in T2DM patients [80].

Both the NRI and IDI were used to compare the diagnostic capabilities of the two indicators by assessing whether one indicator improved the diagnostic accuracy of the other indicator.

NRI quantitatively indicates to what extent the diagnostic accuracy of one indicator is better than the accuracy of another indicator. The calculation method is $NR\hat{I}={\hat{p}}_{up,events}-{\hat{p}}_{down,events}-{\hat{p}}_{up,nonevents}-{\hat{p}}_{down,nonevents}$. The disadvantage is that only the situation of the threshold is considered. IDI can make up for this. IDI uses the model (or indicator) to calculate the prediction probability for each individual. The calculation method is $ID\hat{I}=({\bar{\hat{p}}}_{new,events}-{\bar{\hat{p}}}_{new,nonevents})-({\bar{\hat{p}}}_{old,events}-{\bar{\hat{p}}}_{old,nonevents})$, and the IDI test idea is similar to AUC and considers the overall situation of the indicator under different boundaries, allowing it to represent the overall improvement in the indicator (model). The application of NRI and IDI quantitatively solves the problem of the comparison of the diagnostic efficacy of the two models and can quantitatively evaluate the improvement in the diagnostic efficacy of one indicator over the other. The NRI and IDI indicators can clearly show the proportion of research subjects that are correctly reclassified (discriminated), and they are widely used in research as clinical prediction models.

### Ethics statement

All participants were carefully informed about the protocol and provided written informed consent before inclusion in the study. The study was performed in accordance with the principles of the Declaration of Helsinki.
